# VASP: A Volumetric Analysis of Surface Properties Yields Insights into Protein-Ligand Binding Specificity

**DOI:** 10.1371/journal.pcbi.1000881

**Published:** 2010-08-12

**Authors:** Brian Y. Chen, Barry Honig

**Affiliations:** 1 Department of Biochemistry and Molecular Biophysics, Center for Computational Biology and Bioinformatics, Columbia University, New York, New York, United States of America; 2 Howard Hughes Medical Institute, Columbia University, New York, New York, United States of America; University of Illinois at Chicago, United States of America

## Abstract

Many algorithms that compare protein structures can reveal similarities that suggest related biological functions, even at great evolutionary distances. Proteins with related function often exhibit differences in binding specificity, but few algorithms identify structural variations that effect specificity. To address this problem, we describe the Volumetric Analysis of Surface Properties (VASP), a novel volumetric analysis tool for the comparison of binding sites in aligned protein structures. VASP uses solid volumes to represent protein shape and the shape of surface cavities, clefts and tunnels that are defined with other methods. Our approach, inspired by techniques from constructive solid geometry, enables the isolation of volumetrically conserved and variable regions within three dimensionally superposed volumes. We applied VASP to compute a comparative volumetric analysis of the ligand binding sites formed by members of the steroidogenic acute regulatory protein (StAR)-related lipid transfer (START) domains and the serine proteases. Within both families, VASP isolated individual amino acids that create structural differences between ligand binding cavities that are known to influence differences in binding specificity. Also, VASP isolated cavity subregions that differ between ligand binding cavities which are essential for differences in binding specificity. As such, VASP should prove a valuable tool in the study of protein-ligand binding specificity.

## Author Summary

Proteins carry out vital and specific functions by physically binding other molecules. Understanding specificity, the preferential binding of certain molecules to one another, is essential for numerous medical and industrial applications. Given the structure of a protein with unknown function, algorithms are available that suggest hypothetical functions based on structural similarities to better-studied proteins, even at vast evolutionary distances. In contrast, few algorithms identify structural differences that relate to differences in specificity among closely-related proteins. To address this problem, we present a Volumetric Analysis of Surface Properties (VASP). VASP differs from existing methods because it compares solid representations of protein structures and cavities based on principles from computer graphics and computer aided design. In our results, solid representations enabled VASP to isolate elements of protein structure that create differences in binding sites and thereby lead to differences in binding preferences. These observations point to applications for the annotation and engineering of protein specificity.

## Introduction

The comparative analysis of protein structures is widely used to infer protein function. Geometric alignment of entire structures or of individual domains can reveal that two proteins are related even if this is not evident from sequence. Numerous techniques have been developed for this purpose, most based on either the superimposition of the polypeptide backbone [1]–[5], the comparison of geometric graphs [6], [7] or the alignment of a matrix of distances between individual amino acids [8]. A second type of approach involves the direct comparison of functional sites, such as the geometric disposition of catalytic residues [9]–[13] or the comparison of the shapes of cavities on the protein surface [14]–[18]. Surface representations of proteins [19]–[24] are, in particular, widely used as they reveal shape recognition features that underlie binding specificity. Most approaches reported to date have focused on remote homology detection with the goal of identifying similarities between two or more proteins that can give hints as to biological function. However, a large class of phenomena depend on the ability of closely related proteins to bind similar but non-identical ligands. In such cases the function of a protein as normally defined is well-known but its binding preferences may not be.

**Figure 1:**
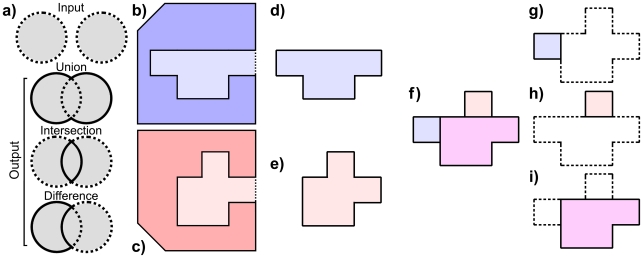
CSG analysis of protein cavities. a) An example of CSG operations showing the borders of input (dotted) and output (solid) regions colored in grey (grey everywhere). b,c) Polygons representing the region occupied by protein **X** (blue) and **Y** (red), shown with molecular surfaces (black lines), and their cavities **x** (light blue) and **y** (light red). The exterior border of each cavity, defined as the convex hull of amino acids lining the cavity, is shown as a dotted line. d,e) **x** (light blue) and **y** (light red) and their borders (black lines), defined by the molecular surfaces and exterior cavity borders of **X** and **Y**. f) Superimposed borders of **x** and **y** (black lines), based on a structural alignment of **X** onto **Y**, the region where the **x** and **y** overlap (magenta), the portion of **x** that does not overlap **y** (light blue), and the portion of **y** that does not overlap **x** (light red). g) A portion of **x** (light blue) that does not overlap **y** (white, dashed outline), h) A portion of **y** (light red) that does not overlap **x** (white, dashed outline). i) Common region of **x** and **y** (magenta), and varying regions (white, dashed outline).

The problem we are specifically addressing concerns the case where two or more proteins have been structurally aligned and it is of interest to identify conserved and varying regions in their binding cavities. Conserved regions, for example, might bind a molecular fragment that is common to substrates acted on by the entire protein family, while the source of differences in intrafamily specificity would likely reside in regions where cavities vary. Our approach is based on a volumetric representation of binding cavities (Figure 1) that is generated with a new program, VASP (Volumetric Analysis of Surface Properties). VASP uses Constructive Solid Geometry (CSG) to compare regions in space defined by a polyhedral boundary [25], [26]. Developed originally for the computer aided design of machine parts [26], and adapted later for computer graphics [25], CSG enables volumetric unions, intersections, and differences of two aligned regions to be computed as if they are solid objects. These CSG operations are a novel tool in the analysis of protein structures because they yield an approximation to the shape of solid regions that is varying or conserved, among protein structures and protein cavities, that is not possible with existing structure comparison methods.

The solid representations used in VASP differ fundamentally from point-based and surface-based representations, which are used in existing methods to define and compare cavities. Point-based representations compare the geometric coordinates of atoms related by one-to-one correspondences. These correspondences cannot be fully constructed between all atoms of sidechains with different lengths, forcing the simplification of sidechain geometry into pseudo-atom or backbone-only representations. In contrast, solid representations compare regions defined by the molecular surface, whose shape reflects the position of any atom without simplification. Solid and surface-based representations both measure differences in molecular shape and curvature. However, surface representations cannot disassemble surface cavities to isolate conserved (intersecting, Figure 1i) or varying (difference, Figure 1g, 1h) regions, as VASP does with CSG, because surface representations do not represent the interior or exterior of a boundary surface. To our knowledge, VASP is the first application of CSG to protein structure comparison, although small molecules have been previously compared in a related manner with lattice points [27] and voxels [28], which are both precursors to Marching Cubes [29], the origin of our technique. These earlier techniques use rectilinear representations that cannot approximate the curvature of molecular surfaces, as VASP does. Other volumetric methods have also been developed to capture topological differences in electrostatic isocontours [30] and to represent regions where substrates overlap for the design of inhibitors that evade drug resistance [31].

The input to VASP includes the definition of binding cavities obtained from manual observation or cavity detection algorithms [16], [22], [32]–[36], and structural alignments of entire proteins [1]–[15], [17], [18]. VASP then uses CSG comparisons of aligned cavity volumes to enable several unique capabilities. Unlike existing methods, VASP can identify individual amino acids and cavity subregions that create structural differences in ligand binding cavities that influence binding specificity. Such functionalities suggest novel applications in protein engineering and design and in the detailed characterization of the determinants of ligand binding specificity. We demonstrate VASP's capabilities with applications to the START domains and to the peptide binding cleft of serine proteases.

## Methods

VASP represents three dimensional regions with a signed field, a mathematical construct that describes every point in space as either inside, outside, or on the surface bordering a given region. We approximate the surface of these regions with Marching Cubes [29], a method first applied to visualize protein surfaces using the GRASP program [23] and also applied widely to visualize magnetic resonance imaging data [37] and electron densities [38]. We use Marching Cubes for the comparison of protein structures and protein cavities because of its compatibility with CSG operations, as described by [25]. We approximate the volume within these regions using a technique called the Surveyor's Formula [39]. In addition to the descriptions below, pseudocode outlining these methods is provided in Text S1 and two optimizations for Marching Cubes are described and benchmarked in Text S4 and Table S1.

### Computing CSG operations with Marching Cubes

**Figure 2:**
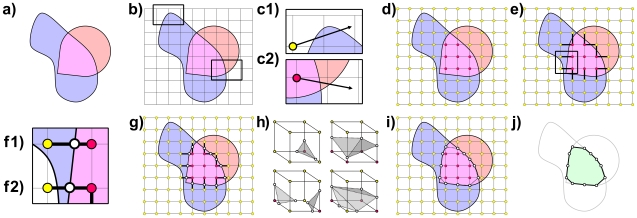
Computing a volumetric intersection using Marching Cubes. a) Input regions A (light blue) and B (light red) with molecular surfaces S_A_ and S_B_ (black lines), and overlapping region (magenta). b) Axis aligned cubic lattice (black grid). Zoomed regions in c1 and c2 (rectangles). c1) A randomly oriented ray intersecting S_A_ twice, emanating from a point (yellow circle) outside A. c2) A randomly oriented ray intersecting both S_A_ and S_B_ once, emanating from a point (red circle) inside the overlapping region. d) Lattice points inside (red) and outside (yellow) the overlapping region, based on ray testing. e) Selected segments (heavy black segments). Zoomed region (black rectangle) illustrated in f1 and f2. f1) Crossing point (white circle) of a selected segment intersecting the triangles of only S_B_. f2) Crossing point (white circle) of a selected segment intersecting the triangles of both S_A_ and S_B_. g) Crossing points (white circles) of all selected segments. h) Four examples from the lookup table that provides triangle layouts (shaded grey triangles, dotted borders) connecting the crossing points (white circles) for cubes with various interior/exterior (red/yellow) lattice point states. i) Two dimensional “triangles” (dotted lines) connecting the crossing points (white circles). j) Approximation of the output region (light green) based on triangles of the output surface (black lines) generated in i.

As input, Marching Cubes requires the desired output resolution, which specifies how finely the output region will be approximated, the desired CSG operation, union, intersection or difference, and two closed regions A and B (Figure 2a), defined by their surface boundaries S_A_ and S_B_, representing, in this work, aligned cavities. The output of Marching Cubes is a region represented by a boundary surface that is approximated with a triangular mesh (Figure 2j).

Using intersection as an example, the overall procedure (Figure 2) is to approximate the shape of the overlapping region (Figure 2a) shared by A and B. First, we construct an axis aligned cubic lattice (Figure 2b) so that, along any dimension, every triangle of A and B is within the bounds of the lattice. We interpret the lattice as a grid of “lattice points,” incrementally spaced along the primary axes according to the desired output resolution, or as a set of “lattice segments” connecting pairs of co-axial lattice points, or as a collection of identically sized “lattice cubes” sharing lattice segments. The lattice is a scaffold for generating the triangles of the output surface.

Second, each lattice point p is determined to be either inside or outside the overlapping region by first testing if p is inside or outside A and B, individually (Figure 2d). We determine if p is inside A by generating a randomly oriented ray originating at p. A is not infinitely large, so the ray must eventually extend outside S_A_, perhaps intersecting the triangles of S_A_ several times. Beginning from the outside, we count these intersections backwards along the ray, crossing into and out of A each time the ray passes through S_A_. Therefore, for an even number of intersections (Figure 2c1), p is outside A. For an odd number of intersections, p is inside A. We apply the same even/odd method to test if p is inside B. If p is inside A and p is inside B, then p must be inside the overlapping region, as illustrated in Figure 2c2. Otherwise, p must be outside the overlapping region.

The third step begins by selecting lattice segments that connect a lattice point inside the overlapping region to a lattice point outside the overlapping region, as shown in Figure 2e. Since the overlapping region of two closed regions must be closed, all selected segments necessarily exit the overlapping region at a “crossing point” p_0_ (Figure 2g) where the selected segment intersects S_A_ or S_B_ or both. If only one of S_A_ and S_B_ intersect the selected segment, as shown in Figure 2f1, or if S_A_ and S_B_ intersect at the same point, then p_0_ is that point of intersection. If S_A_ and S_B_ intersect the selected segment at different points, we call these points p_A_ and p_B_. If p_A_ is inside B, then p_A_ is on the border of A but still inside B, so p_A_ must be at the border of the overlapping region, and thus p_0_ = p_A_. Conversely, if p_B_ is inside A, as shown in Figure 2f2, then, for the same reasons, p_0_ = p_B_.

Finally, we analyze each lattice cube. For each cube, there are 2^8^ = 256 possibilities for the interior/exterior state of its 8 lattice points. Each state corresponds to a unique way for one or more parts of the output surface to pass through the lattice cube, leaving some combination of the lattice points inside or outside the overlapping region. The crossing points indicate precisely where the border of the overlapping region intersects with the lattice segments of the cube. All that remains is to connect the crossing points with triangles to approximate the border of the overlapping region inside the cube, as shown with four examples in Figure 2h. Since there exists 256 different triangular configurations, a lookup table, described elsewhere [29], provides a triangular configuration for every possibility. Notably, the triangles have a directional orientation, defined to face away from the interior of the surface. To denote the orientation of a triangle, a fact we use later, the corners are enumerated in counterclockwise order, when viewed from an exterior perspective. These “output triangles” are depicted as black dotted lines in Figure 2i, since the figure is two dimensional. The output triangles approximate the border of the overlapping region, but are not necessarily identical to the triangles of either S_A_ or S_B_. Proper selection of the output resolution can reduce inaccuracies in the output surface. The final output region (Figure 2j) is within the surface composed by the output triangles.

### Approximating interior volume using the Surveyor's Formula

**Figure 3:**
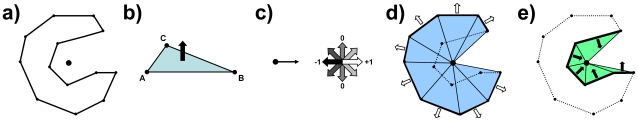
Applying the Surveyor's Formula to measure volume. a) Input region A (white, enclosed), with boundary surface S_A_ and centroid (black dot). b) The normal of a triangle (black arrow) based on the counterclockwise specification of its corners (A,B,C). c) The vector from the centroid to the triangle (thin black arrow, left), several possible normal vectors (thick black, grey, and white arrows), and the resulting dot product (numbers) for different orientations of a given normal. d) Tetrahedra (light blue triangles) based on triangles in S_A_ (thick black lines) with normals (white arrows) facing away from the centroid. S_A_ is shown in dotted lines, for reference. e) Tetrahedra (light green triangles) based on triangles in S_A_ (thick black lines) with normals (black arrows) facing towards the centroid. S_A_ is shown in dotted lines, for reference.

As input, we begin with a closed region A represented by a boundary surface S_A_ composed of oriented triangles. From the input, we compute the centroid c of all triangle corners (Figure 3a). Looping through each triangle t in S_A_, we keep a running total, V, initially zero, of the volume within S_A_, while performing the subroutine below. After all triangles have been considered, the final value of V is the volume within S_A_.

First, we compute the centroid of the triangle, t_c_, and the normal vector of the triangle, t_n_. t_n_ is perpendicular to the plane of t, but for any plane, there are two perpendicular directions. Using the fact that t is oriented, we select t_n_ to point away from the inside of S_A_ (Figure 3b). Second, we determine if t faces away from c or towards c, by measuring the dot product d between t_n_ and the vector (t_c_-c) (Figure 3c). Next, we generate the tetrahedron T, with corners based on the three corners of t, and the global centroid c. We measure the volume of T, v(T), using Tartaglia's rule, described below. If d is positive, we add v(T) to V (Figure 3d), if d is negative, we subtract v(T) from V (Figure 3e). If d is zero, v(T) is also zero, in which case we do nothing and proceed to the next triangle.

Tartaglia's Rule [40] is a three dimensional generalization of Heron's Formula for the area of a triangle [41]. Here, the volume V of a tetrahedron with corners a, b, c, and d, can be evaluated with the expression
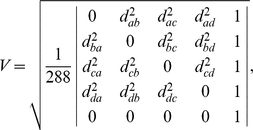
where the distance between two corners x and y is *d*_xy_.

### Converting known functional sites into a volumetric representation for VASP

**Figure 4:**
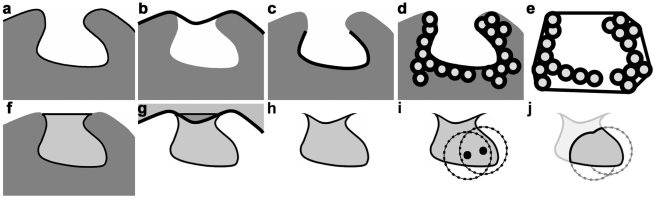
Generating cavity regions. a) Schematic of a protein structure with the area enclosed by the molecular surface (black line) shown in dark grey. b) The envelope surface, defined by a 5 Å, probe sphere, shown with a thick black line. c) The surface of the cavity, shown with the black line, defined as the largest patch of the molecular surface that lies further than 2A from the envelope surface. d) Atoms (circles) belonging to amino acids containing at least one atom that is closest to a triangle on the surface. e) The black line corresponds to the convex hull formed by the Van der Waals radii of the atoms in d. f) The region within the convex hull, defined in e, and outside the molecular surface of the protein is shown in light grey. g) The envelope surface (thick black line), and the region outside the envelope surface (translucent grey). h) The region defined in f that is also inside the envelope surface. i) Two ligand atoms in the cavity (black circles), and spheres defined at a given radius around the atoms (notched lines). j) A subsite (grey, black boundary) defined to be within the cavity in h (faded), and the union of the spheres from i (grey notched lines).

We use SCREEN [35] to identify cavities as input for VASP. SCREEN produces lists of amino acids nearby the cavity, which we convert into a volumetric representation using the procedure illustrated in Figure 4: First, GRASP2 [3] is used to compute triangular meshes approximating the molecular surface based on a 1.4 Å probe (Figure 4a), and an “envelope” surface based on a 5.0 Å probe (Figure 4b). Second, all patches of triangles on the molecular surface with corners further than 2 Å from any location on the envelope surface are identified as the base of each surface cavity (Figure 4c). Third, the patch closest to the amino acids produced by SCREEN is manually selected for the analysis that follows. Fourth, for every triangle in the selected patch, the closest atom in the structure is found and the amino acid it belongs to is added to a non-redundant list. This list contains all amino acids lining the selected patch (Figure 4d). Fifth, the qhull program [42], is used to compute the convex hull of the Van der Waals spheres of the amino acids lining the selected patch (Figure 4e). From the region within the convex hull, the region within the molecular surface is removed using the CSG difference operation (Figure 4f), as is the region outside the envelope surface (Figure 4g). The resulting region (Figure 4h) defines the cavity. Occasionally, small disconnected regions are created in this process. All but the largest, based on surface area, are removed.

In addition to SCREEN, other methods can be used to identify cavities as input for VASP. Cavities described by lists of amino acids, generated with algorithms for cavity detection [33], [35] or local structural comparison [6], [9], [11]–[13], [15], [17], [18], can be converted into volumetric representations with the procedure described above. Cavities described with surfaces [20]–[23], [34], [35], such as the exterior triangles of an alpha shape within a CAST pocket [34], can be converted into volumetric representations by using the surface as if it was selected in Step 3, above.

CSG can also be used to define a subsite of a cavity. First, we follow the procedure described in Figure 4 to represent the entire cavity. Second, we position spheres in the subsite of interest based on the coordinates of bound ligands and select a radius for each sphere that is large enough to overlap the entire subsite (Figure 4i). Third, we compute the CSG union of all the spheres. Fourth, we calculate the intersection between the sphere union and the cavity (Figure 4j). The resulting region defines the shape of the subsite, without including the wider cavity.

GRASP2 surfaces [3], using Van der Waals radii taken from [43], are exceptionally precise approximations of the molecular surface, averaging 384461 triangles per surface, and triangular area averaging .026 Å^2^ on our data set. Some GRASP2 surfaces contain topological discontinuities where single contiguous surfaces are represented with disconnected patches. Input surfaces exhibiting topological discontinuities were first fixed using Polymender [44].

### Volumetric clustering of binding sites

Cavities obtained from a given family of proteins were clustered by “volumetric distance” *V*_(x,y)_,
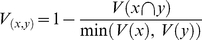
where x and y are cavities, x∩y is the volumetric intersection of x and y, and V(K) represents the volume of a given region K, in Å^3^. The shape of the region x∩y was determined with the CSG intersection, and V(K) was evaluated with the Surveyor's Formula. *V*_(x,y)_ is the proportion of intersecting volume relative to the maximum theoretical degree of intersection, the volume of the smaller region, and thus a measure of volumetric similarity between x and y. We computed *V*_(x,y)_ for all pairs of cavities in each set. Using the “neighbor” tool from Phylip [45], we summarized the overall organization of volumetric conservations and variations using UPGMA clustering (Unweighted Pair Group Method with Arithmetic mean, [46]) of *V*_(x,y)_, over all pairs of cavity regions.

### Clustering other measures of protein similarity

We also clustered proteins in our data set using other metrics of similarity. Multiple sequence alignments were computed with ClustalW 2.0.7 [47] and the most parsimonious phylogeny was constructed with the “protpars” tool from Phylip [45]. Phylogenetic trees generated in this manner are unrooted, so a logical root was selected manually for visual comparison. Backbone structure similarity was computed with Ska [5], and the RMSD of corresponding C_α_ atoms was clustered by UPGMA using the “neighbor” tool from Phylip.

### Identifying amino acids that influence cavity shape

We begin with aligned proteins X and Y, with cavities x and y. First, we generate the molecular surface S_a_ of each amino acid a in X, individually. Second, we compute the CSG intersection between a and y, and measure the volume of the intersection using the Surveyor's Formula. Amino acids with a nonzero volume of intersection cause x to have a different shape than y.

### Identifying volumetrically conserved and varying regions

Regions conserved among aligned cavities are determined by repeated application of CSG intersection. Regions occupied by at least one cavity, among several, are determined with the CSG union. Regions in a cavity x that are not in a cavity y are determined with the CSG difference. For example, the region conserved in all trypsin cavities that overlaps no elastase cavity, illustrated in Figure 9d, is evaluated as the difference between the intersection of all trypsin cavities and the union of all elastase cavities.

### Protein data sets

The Protein DataBank (PDB - 06.15.2008) [48] contains the structures of 28 START domains and 582 serine proteases, from the chymotrypsin, trypsin, and elastase subfamilies. From each set, we removed functionally undocumented and mutant structures and then structures with greater than 90% sequence identity, leaving a non-redundant subset of 11 START domains and 14 serine proteases. Filtering in this order maximized the number of diverse representative structures, identifying START domains and serine proteases averaging 12% and 47% pairwise sequence identity, respectively. Hydrogen atoms, resolved in only four structures in our dataset, were removed for consistency.

The START domains are lipid transporters whose available structures belong to distinct subgroups that have well documented ligand binding specificities [49]. Three proteins in our set exhibit a specific affinity for cholesterols: MLN64 (pdb: 1em2) [50], StarD5 (pdb: 2r55) [49], and StarD4 (pdb: 1jss) [51]. Five others exhibit binding with a wide range of lipids, including fatty acids, cytokinins, and flavonoids [52] and are referred to here as having “broad specificity”. These proteins include allergen-like proteins from birch (pdb: 1bv1), cherry (pdb: 1e09), celery (pdb: 2bk0), yellow lupine (pdb: 1xdf), and mung bean (pdb: 2flh). The remaining functionally characterized proteins in our set include the human phosphatidylcholine transfer protein (pdb: 1ln1), which only binds phosphatidylcholines [53], human ceremide transporter (CERT) (pdb: 2e3m), a highly specific transporter of ceremides of specific lengths [54], and the yeast oxysterol binding protein Osh4 (pdb: 1zht), which prefers oxysterols to cholesterols [55].

### Structure alignment and cavity preparation

Using Ska [5], the START domains were aligned to the major birch allergen (pdb: 1bv1), which was selected randomly. Cavities were defined in the START domains as described above, without subsite definition. The serine proteases were aligned via Ska to bovine gamma-chymotrypsin (pdb: 8gch), because 8gch exhibits a tryptophan bound in the S1 specificity pocket of the larger peptide binding cleft. The S1 pocket was defined with the subsite technique described above. 5 Å spheres were positioned at all tryptophan atoms and at five waters at the bottom of the 8gch S1 pocket. With all S1 pockets aligned onto the S1 pocket of 8gch, the spheres defined the S1 subsite cavity in all serine proteases. Manually placed waters can also be used to define known subsites, but bound waters and substrate provided an objectively defined subsite for demonstration purposes.

Structural alignments of all proteins in our datasets to an individual structure did not create bias in our results. As described in Text S2 and Figures S1, S2, S3, rerunning our results on a realignment to any other dataset member produced no major differences in our results.

### Implementation and performance details

VASP was developed in ANSI C/C++ and compiled on gcc 3.4.6, for 32 and 64 bit ×86 computing platforms. Visualization was implemented using the OpenGL C/C++ library on Windows XP platforms running Intel Xeon, AMD Athlon 64, and Nvidia Geforce 6800 and 7600 chipsets. Experimentation was performed on quad-core Opteron systems with at least 2 gigabytes of random access memory per core. VASP, a single threaded process, used one core and approximately 1 gigabyte of RAM. All results were computed at .5 Å resolution, which produced accurate results with practical runtimes:

CSG operations converting a known functional site into a volumetric representation involved the entire protein structure, and an average of 1.04 million voxels, 384,461 triangles, and 12.8 minutes (1355 voxels/sec). CSG operations computing the intersection of cavities, rather than whole structures, involved an average of 177,490 voxels, 59,677 triangles, and 5.9 minutes of computation (494 voxels/sec). Finally, CSG operations for individual amino acids involved an average of 2,958 voxels, 2,915 triangles, and 2.77 seconds (1068 voxels/sec). START domain cavities generally had much larger volume than serine protease cavities, and CSG runtimes reflected these differences. Additional runtime details are provided in Table S2.

To further clarify the runtime performance of VASP, in the Supporting Materials, we have provided additional performance details describing the runtime of typical CSG operations (Text S3a, Figure S4) and the runtime/accuracy tradeoff at lower resolutions (Text S3b, Figure S5, S6). These observations suggest that .75 Å resolution can also yield reasonable accuracy, though the clustering of START domains was slightly less accurate at this resolution. In the future, adaptive approaches, using oct-trees instead of uniform voxels, and more efficient strategies for assessing the interior/exterior state of a given point, such as those described elsewhere [44], could potentially reduce runtimes and memory usage while maintaining accuracy.

## Results

### START domains

**Figure 5:**
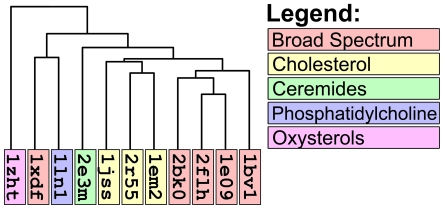
Patterns of volumetric similarity and variation in START domain cavity structure. The topology of the VASP tree clusters START domain cavities based on volumetric distance. The color coding, which is independent of tree topology, indicates the type of ligands that each START domain binds.

Figure 5 reports a clustering of START domains based on volumetric distance. It is evident that the tree separates the 11 proteins into distinct groups that are well correlated with their binding preferences. This separation indicates that VASP is successful in capturing cavity shape similarities and differences among the different proteins that relate to binding preferences. The single outlier in the tree is yellow lupine PR-10 (pdb 1xdf) which is not grouped with other broad specificity START domains. However, 1xdf has a kinked C-terminal helix that fills the ligand binding site and indeed the protein cannot bind ligands in this conformation [56]. Thus, volume-based classification correctly discriminates between 1xdf and the other broad-specificity START domains. It should be noted that global sequence and structure alignment also separated START domains into the correct clusters (Figure S7), but in these cases, 1xdf was included as part of the broad specificity cluster. Thus, global comparisons failed to detect a local change of cavity shape in the binding cavity.

**Figure 6:**
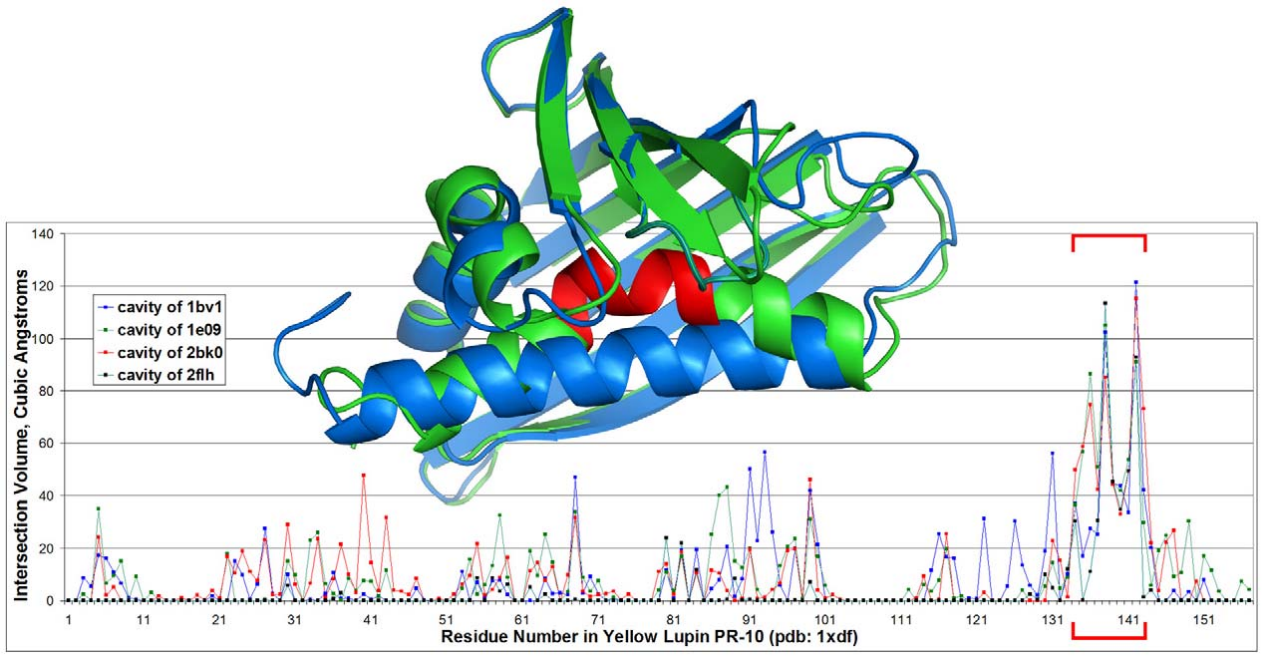
Volumetric intersection of amino acids from yellow lupin PR-10 with other START domains. Each plotted line corresponds to the volume of intersection between the region within the molecular surfaces of the individual amino acids of yellow lupin PR-10 (pdb: 1xdf) and one of the cavities of the other four broad specificity START domains. The red brackets indicate residues 137–144 in 1xdf, which intersect all cavities with high volumes relative to the other amino acids. Inset: structural alignment of 1xdf (green) onto the structure of the major birch allergen (pdb: 1bv1) (blue), rendered with Pymol [65]. Residues 137–144 of 1xdf are shown in red.

We used VASP to identify the regions of the protein responsible for the unusual binding properties of 1xdf. Figure 6 illustrates the degree of volumetric intersection between individual amino acids in 1xdf and the cavities of the other broad-specificity START domains, 1bv1, 1e09, 2bk0, and 2flh. For most amino acids, the volume of intersection averaged 8 Å^3^ (standard deviation 16 Å^3^) over all cavities. That so many amino acids have at least a small degree of overlap is due to the fact that all of these proteins have a very large internal cavity that has some degree of contact with almost every residue. In contrast to this baseline variation, residues 137–144 exhibited unusually high intersection volumes with all cavities considered, averaging 60 Å^3^, with several surpassing 100 Å^3^. These residues are located at the center of the kinked C-terminal helix that fills the binding site of 1xdf and prevents ligand binding (inset, Figure 6). Our ability to identify these residues illustrates how VASP can be used to identify locations in a structure that are responsible for specificity.

### Serine proteases

In serine proteases, affinity for specific sequences of amino acids is associated with individual specificity pockets, S4, S3, .. S1, S1', S2'.. S4', that recognize substrate residues P4, P3, .. P1, P1', P2', .. P4' [57]. In trypsins, S1 exhibits a narrow affinity for amino acids with positively charged side chains [58]; in chymotrypsins, S1 exhibits greatest affinity for large hydrophobic sidechains [59], and in elastases, S1 has greatest affinity for small hydrophobic sidechains [60].

**Figure 7:**
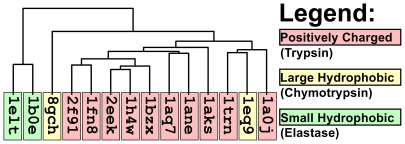
Patterns of volumetric similarity and variation in the S1 specificity pockets of the canonical serine proteases. The topology of the VASP tree clusters serine protease cavities based on volumetric distance. The color coding, which is independent of tree topology, indicates the types of P1 residue preferred by each serine protease.

Figure 7 illustrates the clustering of serine protease S1 pockets based on volumetric distance. Elastase S1 pockets were clustered tightly together and separately from the other serine proteases. With the exception of fire ant chymotrypsin (pdb: 1eq9), trypsins are also clustered tightly together, and separately from other serine proteases. Bovine chymotrypsin (pdb: 8gch) is separated distinctly from the trypsins and from elastases, but also from fire ant chymotrypsin (pdb: 1eq9). Global sequence and structure alignment separated the serine proteases similarly or less well (Figure S8).

**Figure 8:**
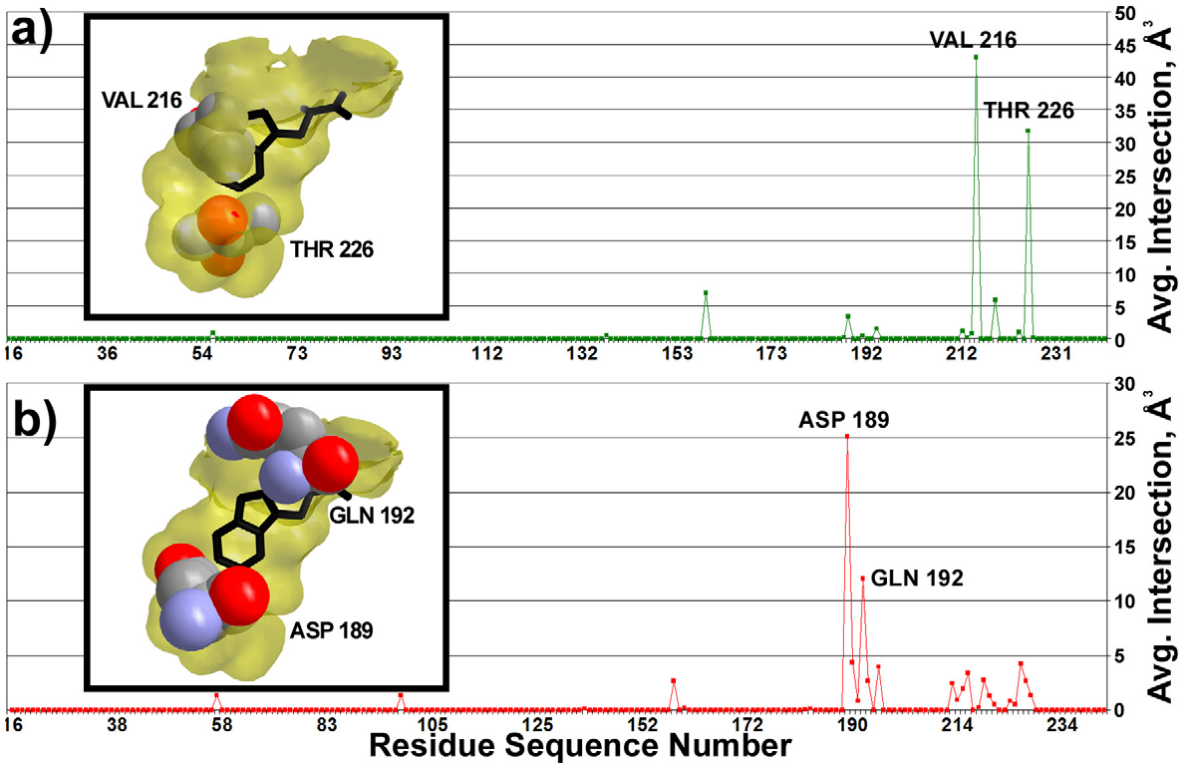
Average volumetric intersections of serine protease amino acids with the cavity of bovine chymotrypsin. a) A plot of the average volume of intersection (Å^3^) between the region within the molecular surface of amino acids at equivalent elastase sequence positions and the cavity of 8gch. Inset: the S1 cavity of 8gch (yellow), a space filling rendition of V216 and T226 (spheres) from Pig Elastase (pdb: 1b0e). b) A plot of the average volume of intersection (Å^3^) between the region within the molecular surface of amino acids at equivalent trypsin sequence positions and the cavity of 8gch. Inset: the S1 cavity of 8gch (yellow), a space filling rendition of D189 and Q192 (spheres) from Salmon Trypsin (pdb: 1a0j). As a visual reference for each inset, the tryptophan bound to the S1 cavity of 8gch is shown in black.

Figure 8 illustrates the degree of volumetric intersection between the individual amino acids of the serine proteases and the S1 cavity of bovine chymotrypsin (pdb 8gch). Intersection volumes were almost always zero or near zero, with a few distinct exceptions: In elastases (Figure 8a), Val216 and Thr226 occupy an average of 43 Å^3^ and 31 Å^3^, respectively, within the 8gch cavity region. These amino acids are known to truncate the S1 pocket (inset, Figure 8a) to generate specificity for small hydrophobic amino acids [61]. In trypsins (Figure 8b), Asp189 occupies an average of 25 Å^3^ within the 8gch cavity and is primarily responsible for the specificity of trypsin for basic residues [62]. Figure 8b illustrates how Asp189 occupies the bottom of the chymotrypsin cavity, which orients the negatively charged carboxylate group of Asp189 to face substrate resides and to sterically hinder the binding of aromatic amino acids. VASP also identifies Glu192, a residue conserved among trypsins that occupies an average of 12 Å^3^ in the 8gch cavity that is not occupied by the Met192 conserved among chymotrypsins. Finally, in fire ant chymotrypsin (pdb: 1eq9) (Figure S9), VASP identifies Asp226, which exhibits a 32 Å^3^ overlap with the bovine chymotrypsin (8gch) cavity. Residue 226 is typically glycine in mammalian chymotrypsins, and, as reported elsewhere [63], Asp226 must rotate out of the way to accommodate the aromatic residues preferred by chymotrypsin.

**Figure 9:**
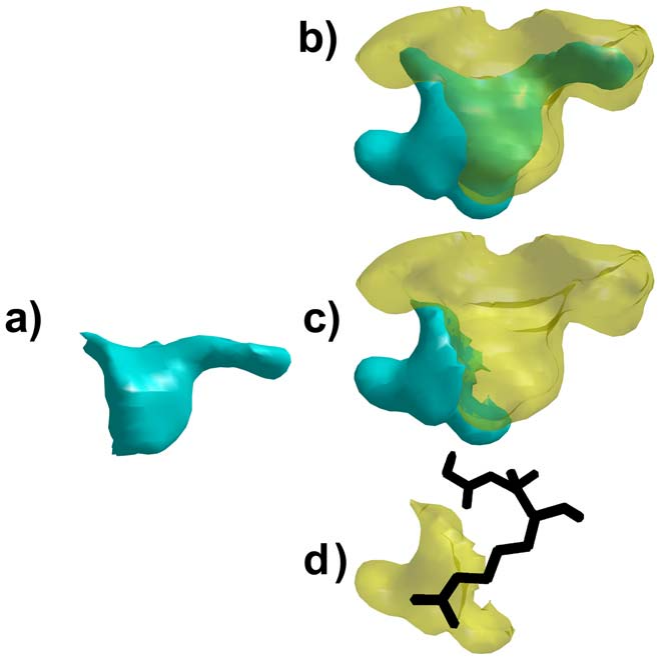
Volumetric decomposition of serine protease S1 cavities. a) The global intersection of all serine protease S1 cavities in our dataset. b) The intersection of all trypsin cavities (teal) and the union of all elastase cavities (yellow). c) The volumetric difference between the intersection of all trypsin cavities and the union of all elastases cavities (teal), and the union of all elastases cavities (yellow). d) The difference between the intersection of all trypsin cavities and the union of all elastase cavities (yellow), and the peptide substrate Gly-Ala-Arg bound to Fusarium oxysporum Trypsin (pdb: 1fn8) (black sticks).

Figure 9 illustrates several regions within the serine protease S1 cavities that are volumetrically conserved or varying. The first region, where all S1 subsites in our dataset overlap (Figure 9a) occupies a volume of 107 Å^3^ and is located at the entrance of the S1 subsite. This global intersection includes a protruding region that extends into the center of the oxyanion hole, a tiny cleft critical for stabilizing hydrolysis reaction intermediates [64]. Only the central portion of the oxyanion hole was conserved among all serine proteases because of slight variations in structural alignments. It is clear that in any serine protease, if any region of the global intersection is obstructed, either P1 would be hindered in entering the S1 cavity or the oxyanion hole would be unable to stabilize reaction intermediates. By determining the global intersection of all S1 cavities, VASP can thus identify functionally significant subregions.

The second region we studied, a 198 Å^3^ volume where all trypsin cavities overlap (Figure 9b) exhibits a distinct 70 Å^3^ protrusion that does not overlap with the region occupied by any elastase cavity (Figure 9c). This conserved cavity protrusion accommodates the longer sidechains bound by trypsin S1 pockets that are occluded by elastase S1 pockets. Figure 9d illustrates one example where the peptide Gly-Ala-Arg, bound to Fusarium oxysporum (pdb: 1fn8), clearly extends its Arginine sidechain into the conserved cavity protrusion. By computing the volumetric difference between the intersection of all trypsins and the union of all elastases, VASP can identify conserved variations between subfamilies of serine proteases that influence specificity for different ligands.

## Discussion

We have presented a new volumetric method for the comparison of protein cavities that is embodied in the VASP program. To our knowledge, VASP is the first program capable of comparing cavities via CSG and it therefore enables a new approach to the characterization of protein binding sites. We demonstrate in an application to START domains that VASP is capable of reproducing known ligand binding specificities and of identifying differences in cavity shapes among proteins that, based on global sequence or structure similarity, might have been expected to be similar. Such differences can result from variations in backbone or sidechain conformation, which are two factors contributing to subtle changes in the shape of binding cavities that would otherwise be hard to detect.

We demonstrate a number of applications of VASP that are not possible with existing methods. One involves the identification of amino acids that contribute to differences in cavity shape. We identified several such amino acids among the START domains and serine proteases and, in each case, reproduced known determinants of ligand binding. A second application is the identification of conserved and varying regions in protein cavities. Among the S1 subsites of the serine proteases, VASP identified conserved regions that are critical for ligand binding, and varying regions that selectively accommodate certain ligands. Overall, we find that VASP creates new opportunities to comparatively analyze and isolate the structural influence of individual elements within protein cavities.

As a first step in the comparison of protein and cavity shape via CSG, VASP exhibits considerable potential for broader applications. When applying VASP more broadly, input structure alignments could include local structure alignments, which would enable proteins with different folds but similar functional sites to enter the analysis. Likewise, as VASP is not a cavity detection algorithm, methods for converting the wide range of cavities detected by existing methods [16], [22], [32]–[35] into a volumetric representation could allow a broader space of input to be analyzed.

VASP has useful applications in contexts where existing protein structure comparison techniques have not been applied. For example, efforts to engineer proteins with altered binding specificities face the practical challenge of being able to test only a few mutants from a combinatorial space of possibilities. By identifying amino acids that influence differences in cavity shape, VASP can suggest a set of mutations to consider. Another possible application is for the annotation of ligand binding specificity on function annotation servers: Given a query protein, function annotation servers can find neighbor proteins with global structure similar to the query. Using VASP, neighbors with bound ligands can be analyzed locally, at their binding sites, to assess volumetric similarity with a known or predicted binding site on the query. Patterns of local volumetric similarity and variation between the query and neighbor might correlate with patterns of ligand binding preferences. Together with other sources of information, volumetric comparison of structurally aligned proteins may thus offer an important tool in protein engineering and function annotation.

## Supporting Information

Figure S1 Volumetric impact of individual amino acids on datset cavities at multiple alignments. a) Volumetric impact of 1xdf residues on broad specificity START domain Cavities at Multiple Alignments. Each line plots the average volume of intersection (vertical axis) of individual residues of 1xdf (horizontal axis) with the cavities of the broad specificity START domains. Different lines correspond to the same computation run with an initial alignment to a different START domain in the dataset. b) Volumetric impact of elastase residues on Chymotrypsin Cavity (8gch) at Multiple Alignments. Each line plots the average volume of intersection (vertical axis) of individual residues of elastases in our dataset (pdb: 1b0e, 1elt, horizontal axis) with the S1 subsite of chymotrypsin (pdb: 8gch). Different lines correspond to the same computation run with an initial alignment to a different serine protease in the dataset. c) Volumetric impact of trypsin residues on Chymotrypsin Cavity (8gch) at Multiple Alignments. Each line plots the average volume of intersection (vertical axis) of individual residues of trypsins in our dataset (pdb: 1a0j, 1aks, 1ane, 1aq7, 1bzx, 1fn8, 1h4w, 1trn, 2eek, 2f91, horizontal axis) with the S1 subsite of chymotrypsin (pdb: 8gch). Different lines correspond to the same computation run with an initial alignment to a different serine protease in the dataset.
(1.22 MB TIF)

Figure S2 Impact of alternate alignments on volumetric clustering of START domain cavities. Clusterings of the START domain cavities computed with initial alignments to different START domains in our dataset. The topology of the VASP tree clusters START domain cavities based on volumetric distance. The color coding, which is independent of tree topology, indicates the type of ligands that each START domain binds.
(3.76 MB TIF)

Figure S3 Impact of alternate alignments on volumetric clustering of serine protease S1 subsites. Clusterings of the serine protease S1 subsites computed with initial alignments to different serine proteases in our dataset. The topology of the VASP tree clusters the subsites based on volumetric distance. For all trees, the color coding, which is independent of tree topology, indicates the preferred P1 residue for each serine protease.
(4.72 MB TIF)

Figure S4 Average runtimes of typical CSG operations at five resolutions. A plot of the runtime (logarithmic, vertical axis) versus the grid resolution (linear, horizontal axis). CSG operations were used in this work for converting known functional sites into a volumetric representation (red line), measuring the pairwise intersection between cavities (green line), and computing the volume of intersection between an individual amino acid and a given cavity (blue line).
(0.16 MB TIF)

Figure S5 Volumetric impact of individual amino acids on dataset cavities, at five resolutions. a) Volumetric impact of 1xdf residues on broad specificity START domain cavities at five resolutions. A plot of the average volume of intersection (Vertical axis) between individual amino acids of yellow lupine PR-10 (pdb: 1xdf, horizontal axis) and the cavities of the broad specificity START domains, computed at five different resolutions (colored lines). b) Volumetric impact of elastase residues on chymotrypsin cavity (8gch) at five resolutions. A plot of the average volume of intersection (Vertical axis) of individual elastase amino acids (pdb: 1b0e, 1elt, horizontal axis) and the S1 subsite of chymotrypsin (pdb: 8gch), computed at five different resolutions (colored lines). c) Volumetric impact of trypsin residues on chymotrypsin cavity (8gch) at five resolutions. A plot of the average volume of intersection (Vertical axis) of individual trypsin amino acids (pdb: 1a0j, 1aks, 1ane, 1aq7, 1bzx, 1fn8, 1h4w, 1trn, 2eek, 2f91, horizontal axis) and the S1 subsite of chymotrypsin (pdb: 8gch), computed at five different resolutions (colored lines).
(0.96 MB TIF)

Figure S6 Impact of reduced resolution on volumetric clustering of dataset cavities. a) Impact of reduced resolution on volumetric clustering of START domain cavities. Clusterings of the START domain cavities computed at five resolutions (.5Å–2.0Å). The topology of the VASP tree clusters START domain cavities based on volumetric distance. The color coding, which is independent of tree topology, indicates the type of ligands that each START domain binds. b) Impact of reduced resolution on volumetric clustering of serine protease S1 subsites. Clusterings of the serine protease S1 subsites, computed at five resolutions (.5Å–2.0Å). The topology of the VASP tree clusters serine protease cavities based on volumetric distance. The color coding, which is independent of tree topology, indicates the types of P1 residues preferred by each serine protease.
(4.92 MB TIF)

Figure S7 Patterns of similarity and variation in the volume, sequence, and backbone structure of START domains. a) The topology of the VASP tree clusters START domain cavities based on volumetric distance. b) The topology of the CLUSTALW tree clusters START domain sequences based on protein sequence identity. c) The topology of the Ska tree clusters START domain backbone geometry based on Å RMSD. For all trees, the color coding, which is independent of tree topology, indicates the type of ligands that each START domain binds.
(2.10 MB TIF)

Figure S8 Patterns of similarity and variation in the volume, sequence, and backbone structure of the canonical serine proteases. a) The topology of the VASP tree clusters serine protease cavities based on volumetric distance. b) The topology of the ClustalW tree clusters serine protease sequences based on protein sequence identity. c) The topology of the Ska tree clusters serine protease backbone geometry based on Å RMSD. For all trees, the color coding, which is independent of tree topology, indicates the preferred P1 residue for each serine protease.
(4.06 MB TIF)

Figure S9 Volumetric intersections of amino acids from fire ant chymotrypsin with the cavity of bovine chymotrypsin. A plot of the volume of intersection (Å^3^) between the region within the molecular surface of the amino acids of fire ant chymotrypsin (pdb: 1eq9) and the cavity of 8gch. Inset: the S1 cavity of 8gch (yellow), spacefilling rendition of Asp 226 (spheres) from 1eq9. As a visual reference, the tryptophan bound to the S1 cavity of 8gch is shown in black.
(0.32 MB TIF)

Table S1 Short rays significantly accelerate VASP performance.
(0.03 MB DOC)

Table S2 VASP performance on START domain and serine protease datasets.
(0.03 MB DOC)

Text S1 Pseudocode describing Marching Cubes and an application of the Surveyor's Formula.
(0.04 MB DOC)

Text S2 On alternative alignments and VASP accuracy.
(0.03 MB DOC)

Text S3 On runtimes, resolution, and accuracy.
(0.03 MB DOC)

Text S4 Optimizing VASP.
(0.03 MB DOC)
