# Supplementary figures and images for: VASP: A Volumetric Analysis of Surface Properties Yields Insights into Protein-Ligand Binding Specificity
Source: PLoS Comput Biol. 2010 Aug 12;6(8):e1000881. doi: 10.1371/journal.pcbi.1000881 (PMC2930297; doi:10.1371/journal.pcbi.1000881)

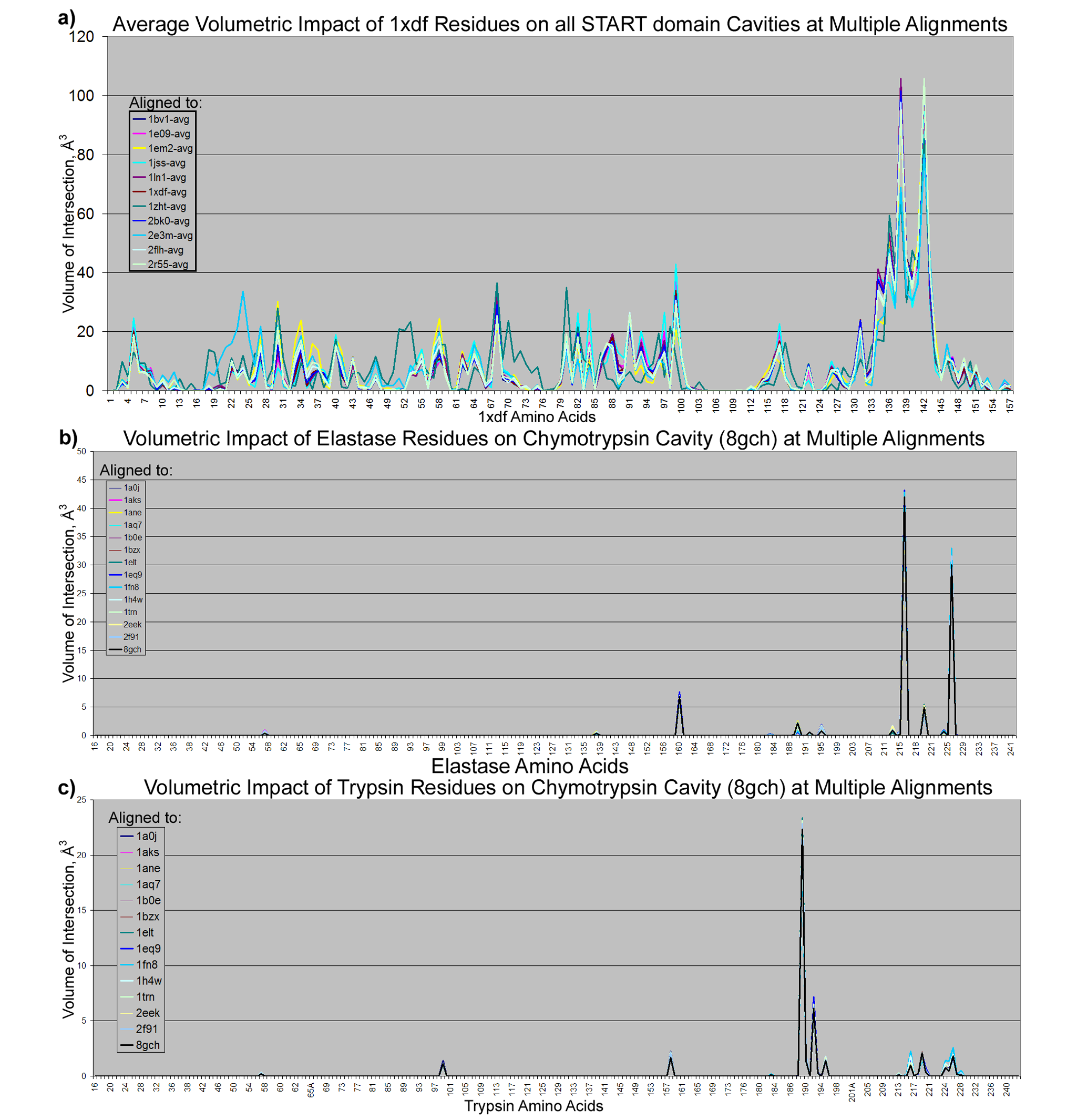

Supplement: Figure S1 — Volumetric impact of individual amino acids on datset cavities at multiple alignments. a) Volumetric impact of 1xdf residues on broad specificity START domain Cavities at Multiple Alignments. Each line plots the average volume of intersection (vertical axis) of individual residues of 1xdf (horizontal axis) with the cavities of the broad specificity START domains. Different lines correspond to the same computation run with an initial alignment to a different START domain in the dataset. b) Volumetric impact of elastase residues on Chymotrypsin Cavity (8gch) at Multiple Alignments. Each line plots the average volume of intersection (vertical axis) of individual residues of elastases in our dataset (pdb: 1b0e, 1elt, horizontal axis) with the S1 subsite of chymotrypsin (pdb: 8gch). Different lines correspond to the same computation run with an initial alignment to a different serine protease in the dataset. c) Volumetric impact of trypsin residues on Chymotrypsin Cavity (8gch) at Multiple Alignments. Each line plots the average volume of intersection (vertical axis) of individual residues of trypsins in our dataset (pdb: 1a0j, 1aks, 1ane, 1aq7, 1bzx, 1fn8, 1h4w, 1trn, 2eek, 2f91, horizontal axis) with the S1 subsite of chymotrypsin (pdb: 8gch). Different lines correspond to the same computation run with an initial alignment to a different serine protease in the dataset. (1.22 MB TIF) [file pcbi.1000881.s001.tif]

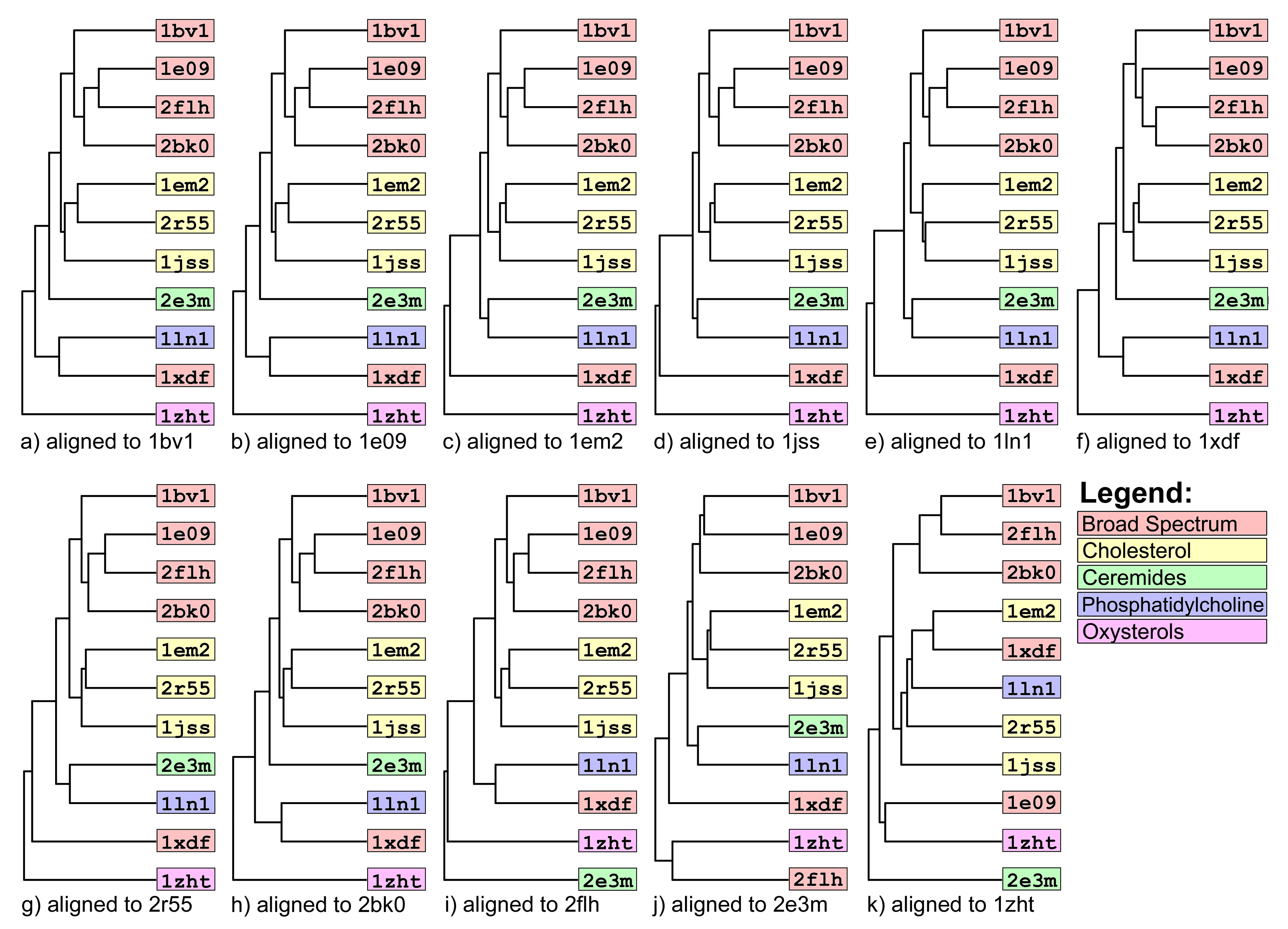

Supplement: Figure S2 — Impact of alternate alignments on volumetric clustering of START domain cavities. Clusterings of the START domain cavities computed with initial alignments to different START domains in our dataset. The topology of the VASP tree clusters START domain cavities based on volumetric distance. The color coding, which is independent of tree topology, indicates the type of ligands that each START domain binds. (3.76 MB TIF) [file pcbi.1000881.s002.tif]

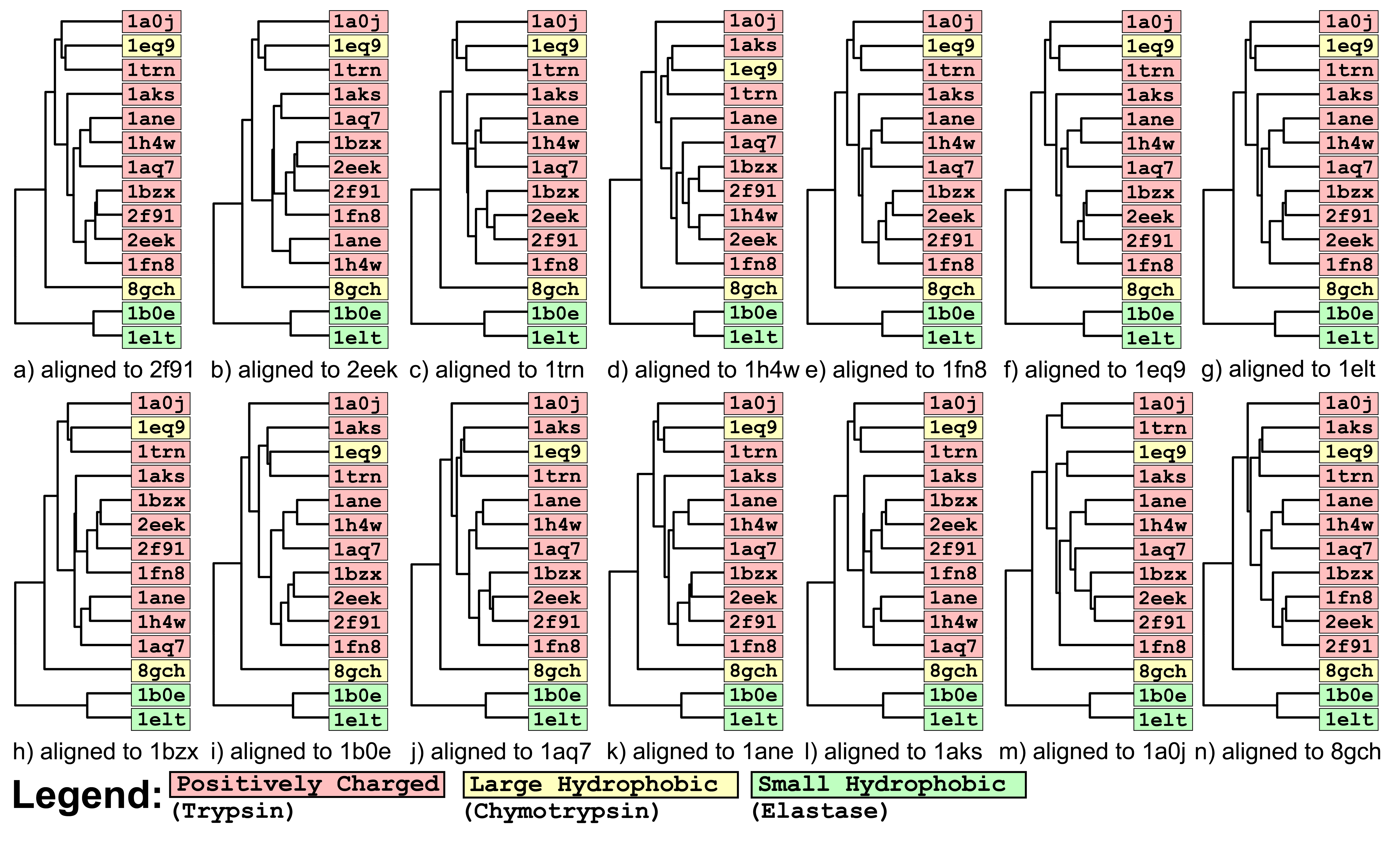

Supplement: Figure S3 — Impact of alternate alignments on volumetric clustering of serine protease S1 subsites. Clusterings of the serine protease S1 subsites computed with initial alignments to different serine proteases in our dataset. The topology of the VASP tree clusters the subsites based on volumetric distance. For all trees, the color coding, which is independent of tree topology, indicates the preferred P1 residue for each serine protease. (4.72 MB TIF) [file pcbi.1000881.s003.tif]

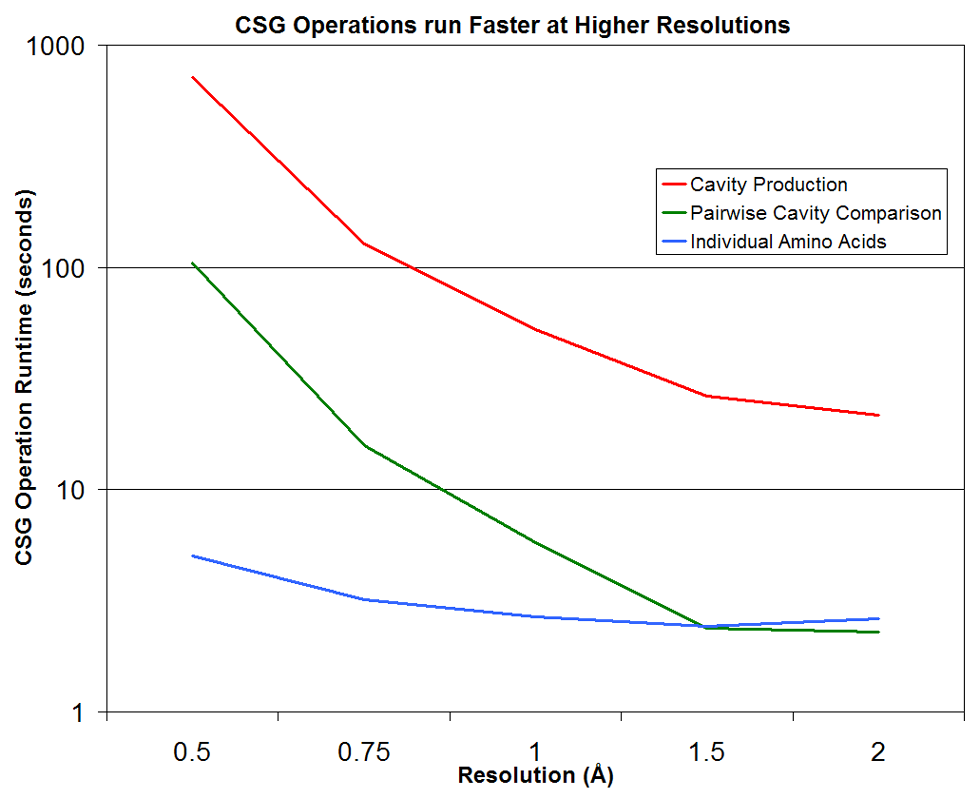

Supplement: Figure S4 — Average runtimes of typical CSG operations at five resolutions. A plot of the runtime (logarithmic, vertical axis) versus the grid resolution (linear, horizontal axis). CSG operations were used in this work for converting known functional sites into a volumetric representation (red line), measuring the pairwise intersection between cavities (green line), and computing the volume of intersection between an individual amino acid and a given cavity (blue line). (0.16 MB TIF) [file pcbi.1000881.s004.tif]

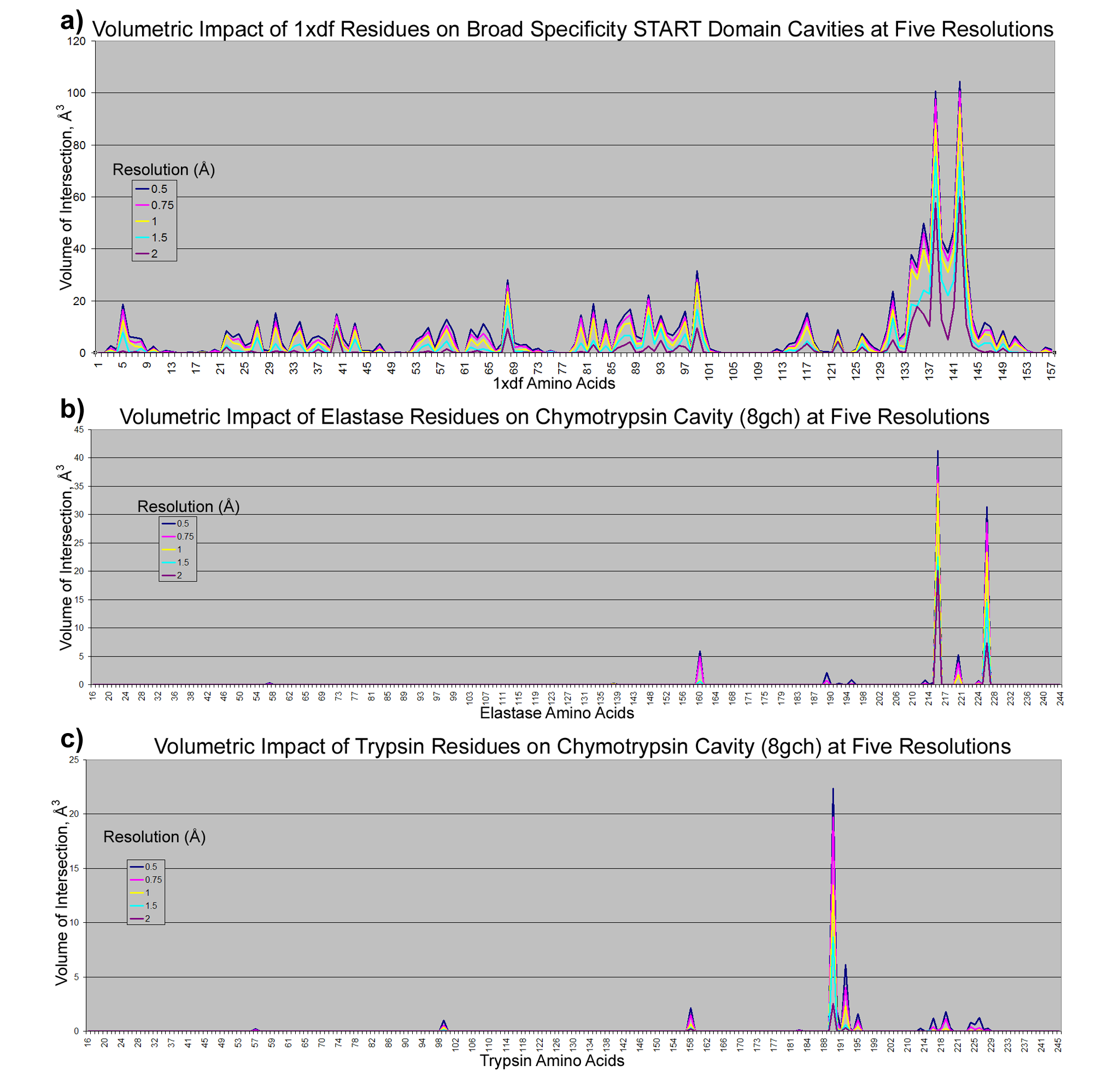

Supplement: Figure S5 — Volumetric impact of individual amino acids on dataset cavities, at five resolutions. a) Volumetric impact of 1xdf residues on broad specificity START domain cavities at five resolutions. A plot of the average volume of intersection (Vertical axis) between individual amino acids of yellow lupine PR-10 (pdb: 1xdf, horizontal axis) and the cavities of the broad specificity START domains, computed at five different resolutions (colored lines). b) Volumetric impact of elastase residues on chymotrypsin cavity (8gch) at five resolutions. A plot of the average volume of intersection (Vertical axis) of individual elastase amino acids (pdb: 1b0e, 1elt, horizontal axis) and the S1 subsite of chymotrypsin (pdb: 8gch), computed at five different resolutions (colored lines). c) Volumetric impact of trypsin residues on chymotrypsin cavity (8gch) at five resolutions. A plot of the average volume of intersection (Vertical axis) of individual trypsin amino acids (pdb: 1a0j, 1aks, 1ane, 1aq7, 1bzx, 1fn8, 1h4w, 1trn, 2eek, 2f91, horizontal axis) and the S1 subsite of chymotrypsin (pdb: 8gch), computed at five different resolutions (colored lines). (0.96 MB TIF) [file pcbi.1000881.s005.tif]

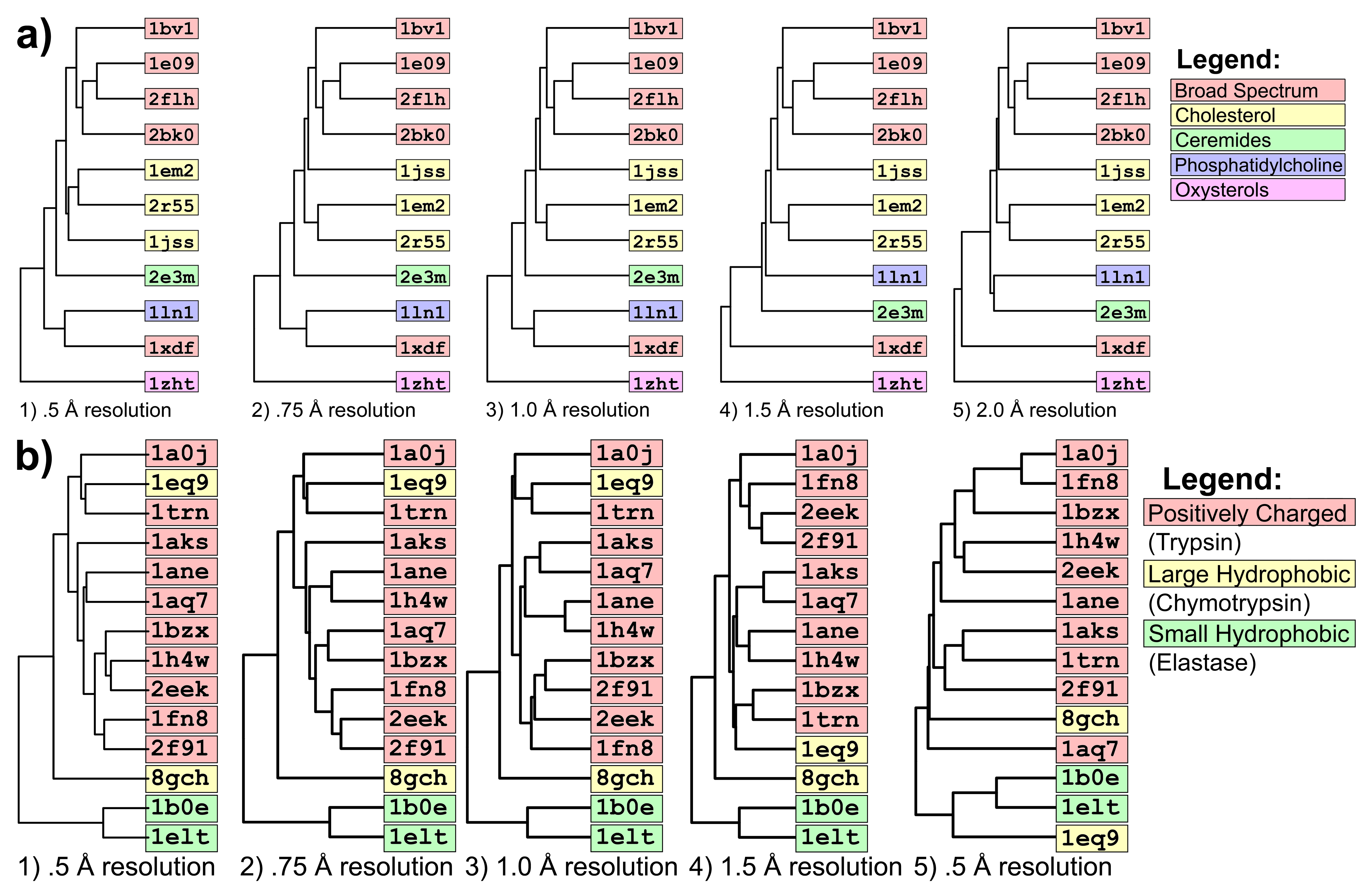

Supplement: Figure S6 — Impact of reduced resolution on volumetric clustering of dataset cavities. a) Impact of reduced resolution on volumetric clustering of START domain cavities. Clusterings of the START domain cavities computed at five resolutions (.5Å–2.0Å). The topology of the VASP tree clusters START domain cavities based on volumetric distance. The color coding, which is independent of tree topology, indicates the type of ligands that each START domain binds. b) Impact of reduced resolution on volumetric clustering of serine protease S1 subsites. Clusterings of the serine protease S1 subsites, computed at five resolutions (.5Å–2.0Å). The topology of the VASP tree clusters serine protease cavities based on volumetric distance. The color coding, which is independent of tree topology, indicates the types of P1 residues preferred by each serine protease. (4.92 MB TIF) [file pcbi.1000881.s006.tif]

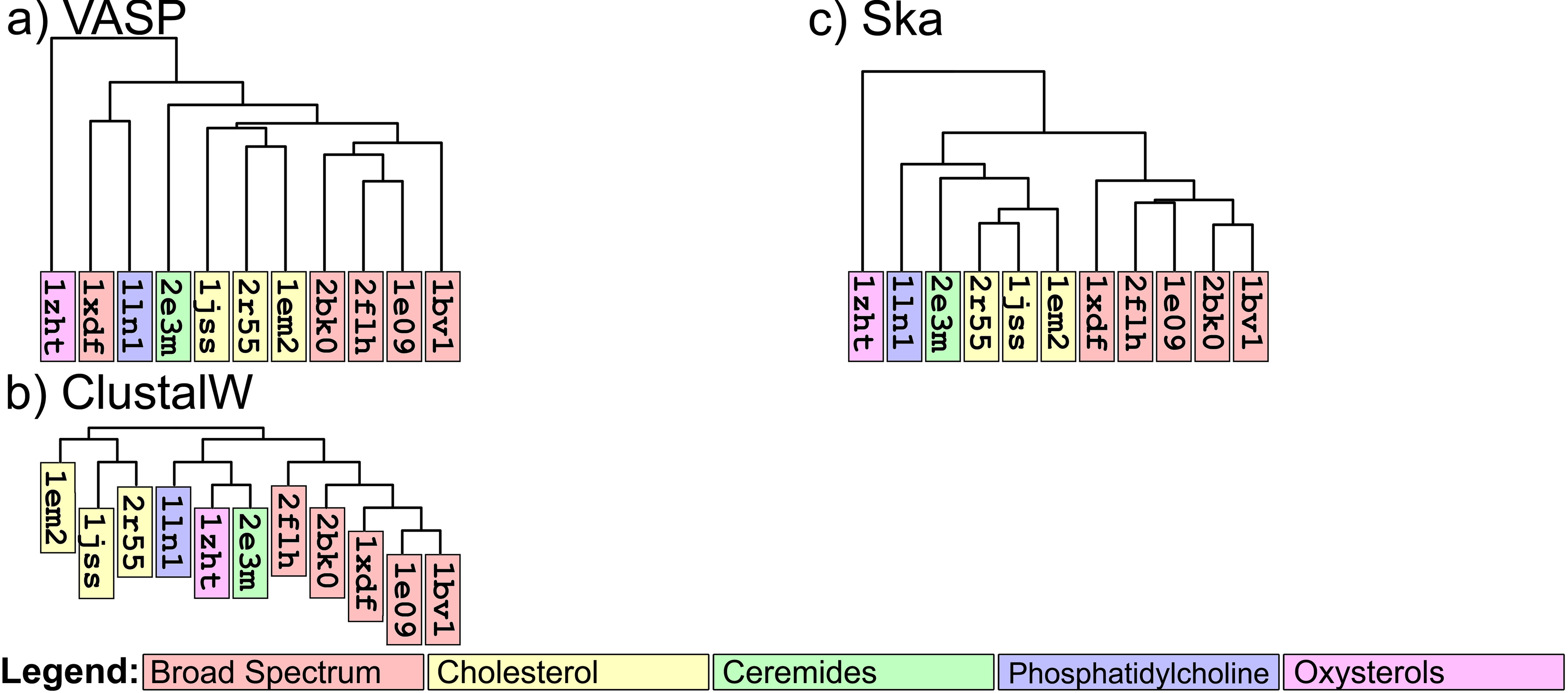

Supplement: Figure S7 — Patterns of similarity and variation in the volume, sequence, and backbone structure of START domains. a) The topology of the VASP tree clusters START domain cavities based on volumetric distance. b) The topology of the CLUSTALW tree clusters START domain sequences based on protein sequence identity. c) The topology of the Ska tree clusters START domain backbone geometry based on Å RMSD. For all trees, the color coding, which is independent of tree topology, indicates the type of ligands that each START domain binds. (2.10 MB TIF) [file pcbi.1000881.s007.tif]

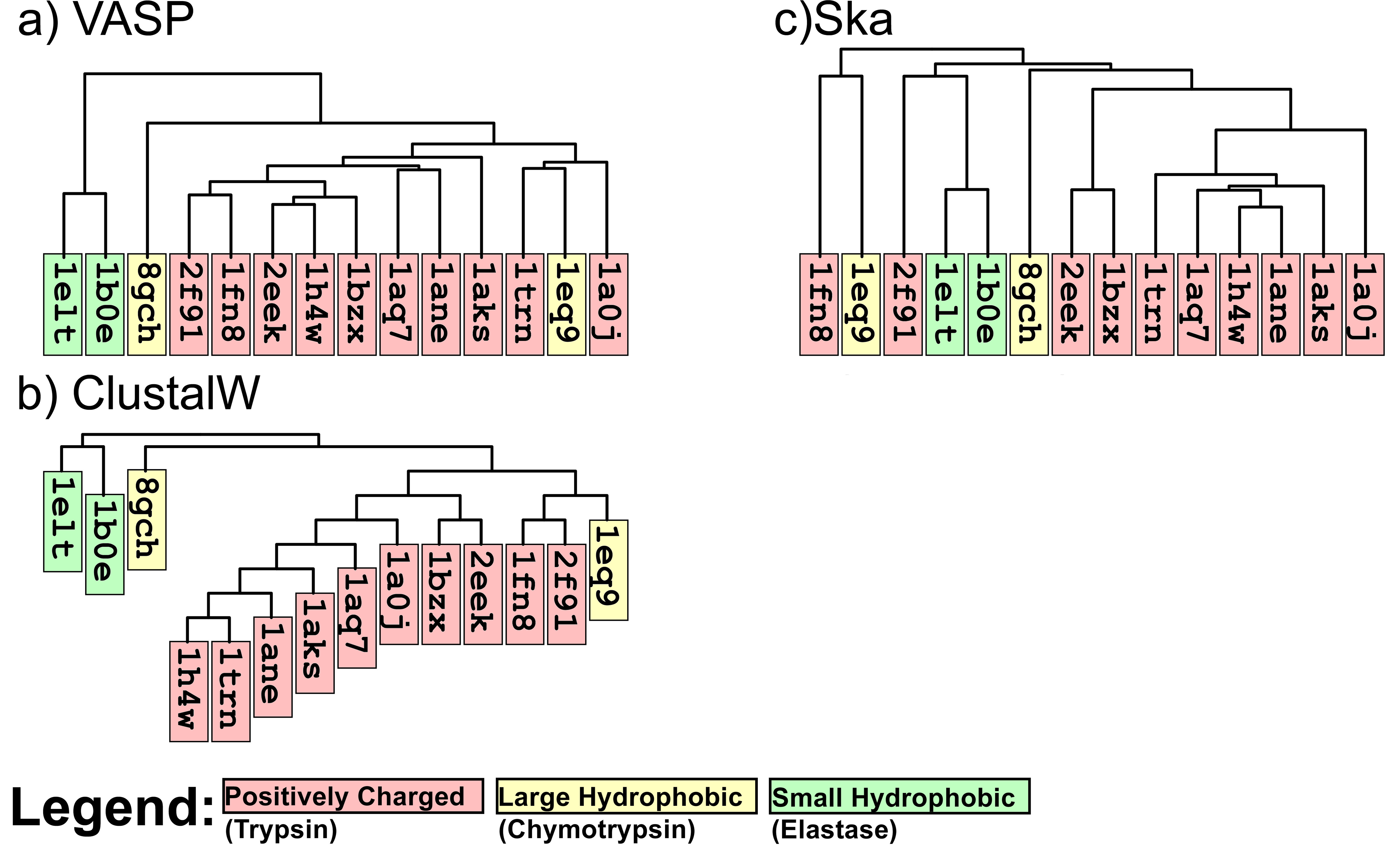

Supplement: Figure S8 — Patterns of similarity and variation in the volume, sequence, and backbone structure of the canonical serine proteases. a) The topology of the VASP tree clusters serine protease cavities based on volumetric distance. b) The topology of the ClustalW tree clusters serine protease sequences based on protein sequence identity. c) The topology of the Ska tree clusters serine protease backbone geometry based on Å RMSD. For all trees, the color coding, which is independent of tree topology, indicates the preferred P1 residue for each serine protease. (4.06 MB TIF) [file pcbi.1000881.s008.tif]

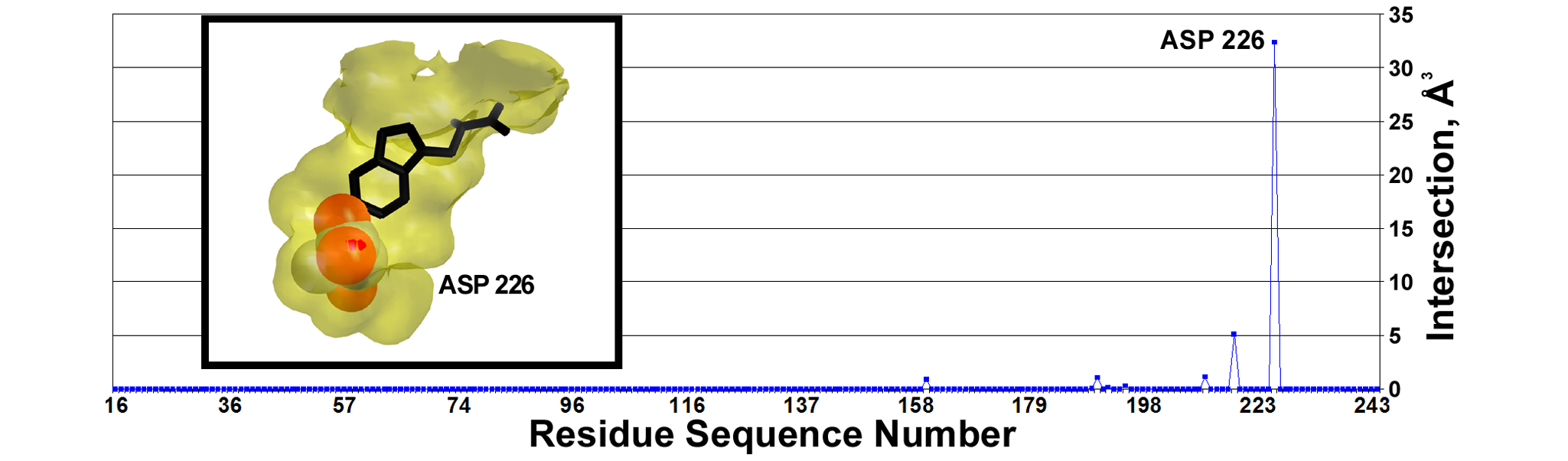

Supplement: Figure S9 — Volumetric intersections of amino acids from fire ant chymotrypsin with the cavity of bovine chymotrypsin. A plot of the volume of intersection (Å3) between the region within the molecular surface of the amino acids of fire ant chymotrypsin (pdb: 1eq9) and the cavity of 8gch. Inset: the S1 cavity of 8gch (yellow), spacefilling rendition of Asp 226 (spheres) from 1eq9. As a visual reference, the tryptophan bound to the S1 cavity of 8gch is shown in black. (0.32 MB TIF) [file pcbi.1000881.s009.tif]
