# Supplementary material for: VASP: A Volumetric Analysis of Surface Properties Yields Insights into Protein-Ligand Binding Specificity
Source: PLoS Comput Biol. 2010 Aug 12;6(8):e1000881. doi: 10.1371/journal.pcbi.1000881 (PMC2930297; doi:10.1371/journal.pcbi.1000881)
Supplement: Table S1 — Short rays significantly accelerate VASP performance. (0.03 MB DOC) [file pcbi.1000881.s010.doc]

Table S1: Short rays significantly accelerate VASP performance

| Average runtimes | Cube Flagging Only | Cube Flagging + Short Rays | Speedup |
| --- | --- | --- | --- |
| Cavity Production | 13,869 sec. (3.8 hrs) | 767 sec. (12.8 min) | 18 |
| Pairwise Cavity Comparison | 2,057 sec. (34.2 min) | 359 sec. (5.9 min) | 5.7 |
| Individual Residue Testing | 9.89 sec. | 2.77 sec. | 3.55 |
