# Supplementary material for: VASP: A Volumetric Analysis of Surface Properties Yields Insights into Protein-Ligand Binding Specificity
Source: PLoS Comput Biol. 2010 Aug 12;6(8):e1000881. doi: 10.1371/journal.pcbi.1000881 (PMC2930297; doi:10.1371/journal.pcbi.1000881)
Supplement: Table S2 — VASP performance on START domain and serine protease datasets. (0.03 MB DOC) [file pcbi.1000881.s011.doc]

Table S2: VASP Performance on START domain and serine protease datasets

| CSG operation (avg. runtime) | START domains | Serine proteases |
| --- | --- | --- |
| Cavity Production | 1530 sec. (25.5 min.) | 243.4 sec. (4.05 min.) |
| Pairwise Cavity Comparison | 982.4 sec. (16.4 min.) | 74.03 sec. (1.23 min.) |
| Individual Residue Testing | 4.39 sec. | 2.43 sec. |
|  |  |  |
| Cumulative Distributed Runtime, all experiments | 90452 sec. (25.1 hrs.) | 23806 sec. (6.61 hrs.) |
