# Supplementary material for: VASP: A Volumetric Analysis of Surface Properties Yields Insights into Protein-Ligand Binding Specificity
Source: PLoS Comput Biol. 2010 Aug 12;6(8):e1000881. doi: 10.1371/journal.pcbi.1000881 (PMC2930297; doi:10.1371/journal.pcbi.1000881)
Supplement: Text S1 — Pseudocode describing Marching Cubes and an application of the Surveyor's Formula. (0.04 MB DOC) [file pcbi.1000881.s012.doc]

**Text S1: Pseudocode**

**a) Marching Cubes**

The pseudocode below describes an application of Marching Cubes for representing the volumetric union, intersection, or difference of two closed triangular meshes. Several subroutines are described as well, for simplicity.

For clarity, one simple optimization was left out of the ***ISINSIDE()*** subroutine: The triangles of the surface ***A*** can be persistently associated with cubes on a uniform grid, and raytesting can first identify the grid cubes that intersect the ray before testing the triangles associated with each cube. This eliminates the need to test many triangles.

**ALGORITHM: Marching Cubes**

**Input:** Closed surface A

**Input:** Closed surface B

**Input:** CSG_OP, one of {UNION, INTERSECTION,DIFFERENCE}

**Input:** Resolution r

let L be an axis-aligned lattice of cubes with sidelength r

let T be an empty list of triangles

**for** each lattice point p in L do

let p_A_ = ***ISINSIDE***(A, p, r)

let p_B_ = ***ISINSIDE***(B, p, r)

let p_OUT_ = ***CSG_STATE***(CSG_OP, p_A_, p_B_)

**end for**

**for** each lattice segment e in L do

let points p1 and p2 be endpoints of e

**if** (p1_OUT_ ≠ p2_OUT_ then)

set edge point p_0_ = ***INT_POINT***(CSG_OP, A, B, p1, p2)

**end if**

**end for**

**for** each cube c in L do

let P = {p0, p1, . . . , p7} be corners of c

let E = {e0, e1, . . . , } be edge points of any segment of c

let triangles t = LOOKUP(P, E)

append t to T

**end for**

**Output:** T, the output boundary surface

***ISINSIDE***(A, p, r)

**Input:** Closed surface A

**Input:** point p

let R be a randomly oriented ray originating at p

let L be an axis-aligned lattice of cubes with sidelength r

let pointList be an empty list of points

**for** each triangle t in A that intersects R do

let p_i_ be the point of intersection between t and R

**if** p_i_ is not already an element of pointList then

add p_i_ to pointList

**end if**

**end for**

**if** the size of pointList is even then

**Output:** false, p is outside A

**else**

**Output:** true, p is inside A

**end if**

***CSG_STATE***(CSG_OP, p_A_, p_B_)

**Input:** CSG_OP, one of {UNION, INTERSECTION,DIFFERENCE}

**Input:** p_A_, true if p is inside surface A, false otherwise.

**Input:** p_B_, true if p is inside surface B, false otherwise.

let the bool result = false

**if** CSG_OP = UNION and p_A_ = true or p_B_ = true then

set result = true

**end if**

**if** CSG_OP = INTERSECTION and p_A_ = true and p_B_ = true then

set result = true

**end if**

**if** CSG_OP = DIFFERENCE and p_A_ = true and p_B_ = false then

set result = true

**end if**

**Output:** result

***INT_POINT***(CSG_OP, A, B, p1, p2)

**Input:** CSG_OP, one of {UNION, INTERSECTION,DIFFERENCE}

**Input:** the surfaces A, B

**Input:** p1, p2, endpoints of the lattice segment e.

Either p1 is p2 is inside the output surface.

Assume without loss of generality that p1 is inside the output surface.

let p_a_ be the point where e intersects A

let p_b_ be the point where e intersects B

**if** either p_a_ or p_b_ does not exist then

**Output:** p_b_ or p_a_, the one that does exist.

**else**

**if** CSG_OP = UNION then

**Output:** p_a_ or p_b_, whichever is further from p1.

**end if**

**if** CSG_OP = INTERSECTION or DIFFERENCE then

**Output:** p_a_ or p_b_, whichever is closer to p1.

**end if**

***LOOKUP***(P, E)

**Input:** P, the inside/outside state of the 8 lattice cube corners.

**Input:** E, intersection points on lattice segments of the cube.

See reference [29] for the lookup table that generates triangles for every cube state.

**Output:** Triangles t that approximate the output surface in the cube.

**b) Surveyor's Formula**

The pseudocode below describes an application of The Surveyor's Formula for computing the volume of the region within a closed triangular mesh.

**ALGORITHM: Surveyor’s Formula**

**Input:** Triangular Polyhedral boundary S_A_

let c be the centroid of all points in S_A_

let V = 0

**for** each triangle t in S_A_ do

let the corners of t = {t_1_, t_2_, t_3_}

let tetrahedron T = {t_1_, t_2_, t_3_, c}

let t_c_ be the centroid of t

let t_n_ be the normal of t, which faces away from the interior of S_A_

let d = DOT_PRODUCT(tn, tc − c), the standard dot product

**if** d < 0 then

set V = V − v(T)

**end if**

**if** d > 0 then

set V = V + v(T)

**end if**

**end for**

**Output:** V, the volume within SA
