# Supplementary material for: VASP: A Volumetric Analysis of Surface Properties Yields Insights into Protein-Ligand Binding Specificity
Source: PLoS Comput Biol. 2010 Aug 12;6(8):e1000881. doi: 10.1371/journal.pcbi.1000881 (PMC2930297; doi:10.1371/journal.pcbi.1000881)
Supplement: Text S2 — On alternative alignments and VASP accuracy. (0.03 MB DOC) [file pcbi.1000881.s013.doc]

**Text S2: On Alternative Alignments and VASP Accuracy**

Our data were computed with an initial backbone alignment of all dataset structures to a single input structure. To understand the influence of the initial alignment on our results, we realigned all structures and cavities to every possible initial alignment, and exhaustively recomputed our results.

Initial alignments based on other protein structures had a very minor impact on the identification of individual amino acids that alter the shape of protein cavities. Figure S1a plots the volume of intersection between individual amino acids of the yellow lupine PR-10 (pdb: 1xdf) and the cavities of the broad specificity START domains at different initial alignments. In every case, the same amino acids, ranging from 137-144, exhibited a large volume of intersection with the broad specificity cavities. The intersection volumes of the remaining amino acids were very similar for all alignments, except when all structures were initially aligned to yeast oxysterol binding protein Osh4 (pdb: 1zht). This structure exhibits a rather different backbone structure from the other START domains, and the resulting alignments appear to be skewed slightly. Figures S1b and S11c plot the volume of intersection between individual amino acids of the elastases and trypsins, respectively, with the S1 subsite of chymotrypsin (pdb: 8gch). In both cases, different initial alignments created few distinguishable differences in the cavity intersection volumes of individual amino acids.

Initial alignments based on other protein structures had minor impacts on the clustering of cavities based on volumetric distance. Alternative alignments of START domains (Fig. S2) produced nearly identical clusterings whose topology reflected patterns in known binding preferences, with two exceptions. An alignment based on human CERT (pdb: 2e3m) produced an incorrect classification of 2flh, and an alignment based on 1zht produced an incorrect classification of 1ln1 and 1e09. Alternative alignments of the serine proteases (Fig. S3) produced clusterings whose topology uniformly relates to known binding preferences, except with the consistent misclassification of 1eq9, which was discussed earlier.

These data suggest that reasonably diverse sets of proteins can be analyzed with VASP without major inaccuracies caused by the initial alignment. However, considering the case of alignments to 1zht, it is clear that proteins with sufficiently different backbone structures can interfere with the initial alignment. While the degree of impact of differing folds is a point to be assessed in future work, it is important to mention that initial alignments do not have to depend on backbone structure. VASP is entirely independent of initial alignments, so the input alignment can therefore be based on local structural alignments, or even manual alignments. While VASP was designed with the goal of analyzing proteins with similar functions and varying specificities, considering alignments based on different criteria could potentially broaden the applicability of VASP.
