# Supplementary material for: VASP: A Volumetric Analysis of Surface Properties Yields Insights into Protein-Ligand Binding Specificity
Source: PLoS Comput Biol. 2010 Aug 12;6(8):e1000881. doi: 10.1371/journal.pcbi.1000881 (PMC2930297; doi:10.1371/journal.pcbi.1000881)
Supplement: Text S3 — On runtimes, resolution, and accuracy. (0.03 MB DOC) [file pcbi.1000881.s014.doc]

**Text S3a: On Coarsening Resolutions and Runtime**

CSG operations were applied for several distinct tasks in our results. These tasks included converting known functional sites into a volumetric representation (red line, Fig. S4), measuring the pairwise intersection between cavities (green line, Fig. S4), and computing the volume of intersection between an individual amino acid and a given cavity (blue line, Fig. S4). The major influence on runtimes for these tasks is the number of voxels to be analyzed. Figure S4 demonstrates that a coarsening resolution can decrease runtimes significantly.

**Text S3b: On Coarsening Resolutions and VASP Accuracy**

Results computed for this paper were recomputed at five coarser levels of resolution, to understand the impact of reduced resolution on VASP Accuracy.

In the analysis of individual amino acids (Fig. S5a, S5b, S5c), as resolution decreased, the volume of intersection with the given cavity diminished for all amino acids. The vast majority of amino acids had no volume of cavity intersection at any resolution. Amino acids with conspicuous volumes of intersection at high resolutions exhibited significantly decreased intersection volumes at lower resolutions. As a result, the significance of the amino acids with the highest volume of cavity intersection became unclear at coarser resolutions.

Figure S5a plots the volumetric intersection of amino acids from the yellow lupin PR-10 (pdb: 1xdf) with the cavities of broad specificity START domains. The amino acids that intersect the cavities most, between residues 137-144, had intersection volumes over 100 Å^3^, when evaluated at .5 Å resolution, but when re-evaluated at 2.0 Å resolution, the same amino acids had less than 60 Å^3^. Figures S5b and S5c plot the volumetric intersection of amino acids in the elastases and trypsins, respectively, with the cavity of 8gch, at multiple resolutions. Among elastases, volumes of cavity intersection for V216 and T226, the two most conspicuous residues, fell from 43 Å^3^ and 31 Å^3^, at .5 Å resolution, to 20 Å^3^ and 7 Å^3^, at 2.0 Å resolution. Among trypsins, volumes of cavity intersection for D189, the most conspicuous residue, fell from 25 Å^3^ to below 5 Å^3^. At higher resolutions, the significance of the volumetric intersection between individual amino acids and a given cavity becomes less clear.

When clustering cavities based on volumetric distance (Fig. S6a, S6b), as resolution decreased, the topology of the clustering reflected known patterns of ligand binding preferences less accurately. Figure S6a illustrates a clustering of the START domain cavities. Here, at .75 Å resolution, we can see that the cavity of 1jss has smaller volumetric distance to the cavities of the broad specificity START domains, rather than to the other cholesterol-binding START domains. This change, away from a topology that reflects binding preferences, persists as resolution coarsens. Figure S6b illustrates a clustering of the serine protease S1 subsites. As resolution coarsens, the topology continues to reflect binding preferences, but significant topological changes occur in the internal clustering of trypsin cavities. The clustering becomes inaccurate at 2.0 Å resolution. Clustering based on volumetric distance ceased reflecting binding preferences at increasing but different resolutions.

These data suggest that the .5 Å resolution used in our experimentation was sufficient for our data. However, it is also clear that more relaxed resolutions can be sufficient in certain applications. In the future, and for different applications, additional investigation into the resolution required for accurate results is necessary.
