# Supplementary material for: VASP: A Volumetric Analysis of Surface Properties Yields Insights into Protein-Ligand Binding Specificity
Source: PLoS Comput Biol. 2010 Aug 12;6(8):e1000881. doi: 10.1371/journal.pcbi.1000881 (PMC2930297; doi:10.1371/journal.pcbi.1000881)
Supplement: Text S4 — Optimizing VASP. (0.03 MB DOC) [file pcbi.1000881.s015.doc]

**Text S4: Optimizing VASP**

When computing a CSG operation in VASP, efficiency can be achieved by rapidly determining whether a given point p is inside or outside an input region. Here, we describe two optimizations that reduce the number of points that must be tested and test the remaining points more rapidly.

Cube Flagging:

For each cube in the cubic lattice, we allow a flag to be set as either INTERIOR or EXTERIOR. All cubes are initially unflagged. Below, we refer to cubes that do not contain part of the border of the input region as “empty cubes”. Empty cubes must be entirely interior or entirely exterior to the input region.

We follow the Marching Cubes procedure normally, except that we do not perform interior/exterior testing on points in flagged cubes. If we test a lattice point p in an unflagged empty cube, we flag that cube as either INTERIOR or EXTERIOR, to agree with the interior/exterior status of p. Before we move on to the next lattice point, using recursive propagation, we apply the same flag to all empty cubes that are part of any sequence of adjacent empty cubes that ends with the cube we flagged. Using this method, a large connected region of empty cubes can be flagged after one test.

Any lattice point on any cube flagged by this process has the same interior/exterior status as p: A line can be drawn from that point, through adjacent empty cubes, to p without crossing the boundary of the input region. As a result, many fewer points must be tested.

Interior/exterior testing with short rays:

As described in the Methods section, interior/exterior checking is achieved by counting the number of times a randomly oriented ray intersects the border of an input region. In order for the test to be accurate, the ray must be long enough that it intersects the boundary of the cubic lattice. Here, we refer to these rays as “long rays”. Finding all the triangles that a long ray intersects can be time consuming, especially if it passes through much of the input region.

Once cube flagging is implemented, however, tests with long rays only need to be used occasionally. For a point p to be tested for interior/exterior status, we can first determine if there exist any flagged cubes nearby. If not, we test with a long ray, but if so, we then simply draw a “short ray” from p to the center of the closest flagged cube. Counting the number of times the short ray intersects the border of the input region, we can then determine the interior/exterior status of p, using the interior/exterior status of the flagged cube. For example, if the cube is flagged INTERIOR, then an odd number of border intersections would make p an exterior point. Since most cubes are nearby an empty, flagged cube, the number of triangles that must be considered for intersection with the short ray is significantly smaller than when testing with the long ray, so the test is faster.
